# Alteration of Occlusal Plane in Orthognathic Surgery: Clinical Features to Help Treatment Planning on Class III Patients

**DOI:** 10.1155/2018/2495262

**Published:** 2018-05-09

**Authors:** Daniel Amaral Alves Marlière, Tony Eduardo Costa, Saulo de Matos Barbosa, Rodrigo Alvitos Pereira, Henrique Duque de Miranda Chaves Netto

**Affiliations:** ^1^Division of Oral and Maxillofacial Surgery, Piracicaba Dental School, State University of Campinas, 13414-903 Piracicaba, SP, Brazil; ^2^Division of Dentistry, Faculty of Medical Science and Health – SUPREMA, 36033-003 Juiz de Fora, MG, Brazil; ^3^Division of Dentistry, Faculty São Leolpoldo Mandic – SLM, 13045-755 Campinas, SP, Brazil; ^4^Department of Oral and Maxillofacial Surgery, Pedro Ernesto University Hospital, State University of Rio de Janeiro, 20551-030 Rio de Janeiro, RJ, Brazil; ^5^Department of Clinical Dentistry, Juiz de Fora Dental School, Federal University of Juiz de Fora, 36036-300 Juiz de Fora, MG, Brazil

## Abstract

Dentofacial deformities (DFD) presenting mainly as Class III malocclusions that require orthognathic surgery as a part of definitive treatment. Class III patients can have obvious signs such as increasing the chin projection and chin throat length, nasolabial folds, reverse overjet, and lack of upper lip support. However, Class III patients can present different facial patterns depending on the angulation of occlusal plane (OP), and only bite correction does not always lead to the improvement of the facial esthetic. We described two Class III patients with different clinical features and inclination of OP and had undergone different treatment planning based on 6 clinical features: (I) facial type; (II) upper incisor display at rest; (III) dental and gingival display on smile; (IV) soft tissue support; (V) chin projection; and (VI) lower lip projection. These patients were submitted to orthognathic surgery with different treatment plannings: a clockwise rotation and counterclockwise rotation of OP according to their facial features. The clinical features and OP inclination helped to define treatment planning by clockwise and counterclockwise rotations of the maxillomandibular complex, and two patients undergone to bimaxillary orthognathic surgery showed harmonic outcomes and stables after 2 years of follow-up.

## 1. Introduction

The exact prevalence of significant dentofacial deformities (DFD) that requires orthognathic surgery as a part of definitive treatment is not quite clear [1]. However, it was estimated that about 5% of the UK or USA population present with DFD that had needed orthognathic surgery as a part of their definitive treatment [2, 3]. Among the DFD, the most prevalent was Class III malocclusion [1], who had been shown in similar studies by findings of several samples in Brazil [4], Saudi Arabia [5], Hong Kong [6], UK [6, 7], Norway [8], and the USA [9].

An index of orthognathic functional treatment needs (IOFTN) had been developed to objectively identify patients that seemed to need orthognathic surgery with low- or high-priority treatments. This index has 5 categories, from a very great need (score 5) through to no need for treatment (score 1), there being patients with scores 4 or 5 had more priority treatment [10]. Borzabadi-Farahani et al. [11] assessed retrospectively the functional needs using the index in DFD patients who had undergone orthognathic surgery. The most Class III patients had presented score 5 of the index that was higher percentages than other malocclusion indicating a functional need for orthognathic surgery.

Regardless of the malocclusion classification, prevalence or priority treatment, Class III patients can present different facials features that will be correlated with cephalometric aspects, one of them is the occlusal plane (OP) angulation [12]. The OP angle is defined as the angle formed by the Frankfort horizontal plane and the line tangent to the canine tips of the lower premolars and the buccal groove of the second molars. The normal value for adults is 8° (±4°). DFD are often related to an abnormal OP angulation, and surgical alteration of this angle may have a substantial impact on the functional and esthetic outcomes for patients [12].

Class III patients may present two facial types correlated to the angulation of the OP and can be highlighted brachycephalic with low OP (<4°) and dolichocephalic with high OP (>12°). The low OP facial type presents with the following characteristics: decreased OP angle; low mandibular plane angle; prominent mandibular gonial angles; strong chin relative to the mandibular dental alveolus; and Class I, Class II, or occasionally, Class III malocclusion. The HOP facial type presents with the following basic characteristics: increased OP angulation; anterior vertical maxillary hyperplasia and/or posterior vertical maxillary hypoplasia; anteroposterior mandibular hypoplasia; high mandibular plane angulation; and Class I, Class II, or Class III malocclusion with or without an anterior open bite [12].

One of the ways to benefit Class III patients such aesthetically as functionality is performed bimaxillary orthognathic surgery by means of treatment plannings based on alterations of the OP [13, 14]. Thus, Class III patients with different angulations of the OP can benefit from rotations of counterclockwise and clockwise of the maxillomandibular complex (MMC) [13]. In this sense, Marlière et al. [13] and Parente et al. [14] showed three clinical cases in Class III patients with different facial types and clinical features that performed different treatment planning in orthognathic surgery by counterclockwise and clockwise rotations of the MMC, but the authors disclosed to be more important the evaluation of the clinical features than clearly the obtainment of OP angulation during treatment planning.

In these case reports, two Class III patients with different clinical features and inclination of OP were presented, undergone different treatment planning, and submitted to alteration of OP by clockwise and counterclockwise rotations of the MMC for orthognathic surgery correction of DFD.

## 2. Case Reports

Two patients presented to the Oral and Maxillofacial Surgery Clinic of the University Hospital Pedro Ernesto (State University of Rio de Janeiro, Brazil) for treatment of dentofacial deformity, which complained of esthetic maxillary deficiency and functional masticatory restrictions. Patients underwent clinical examination (facial analysis and intraoral evaluation) associated with photographs and plaster models of dental arches. The patient signed an informed consent form for both treatment and use of images for publication.

### 2.1. Patient I

**Figure 1:**
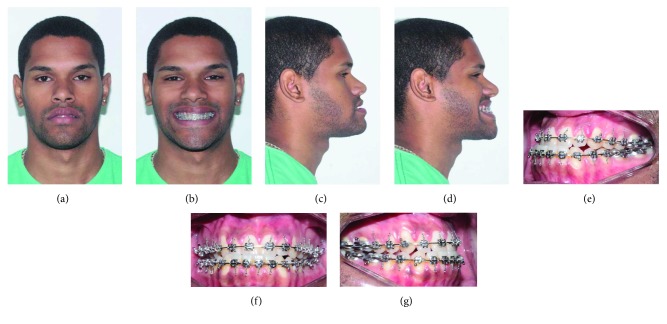
(a–d) Preoperative evaluation at rest and smiling. (e–g) Intraoral images.

A healthy 25-year-old male was undergone to facial analysis which showed brachycephalic morphologic type and Class III malocclusion (Figures 1(a)–1(g)). The clinical features and cephalometric measures (McNamara Analysis) were presented in Table 1.

### 2.2. Patient II

**Figure 2:**
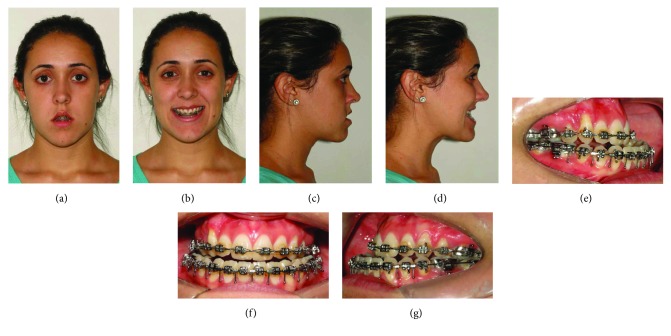
(a–d) Preoperative evaluation at rest and smiling. (e–g) Intraoral images.

A healthy 27-year-old female was undergone to facial analysis which showed dolichocephalic morphologic type and Class III malocclusion (Figures 2(a)–2(g)). The clinical features and cephalometric measures (McNamara Analysis) were presented in Table 1.

#### 2.2.1. Treatment Planning

The treatment planning was aided by clinical examination (facial analysis and intraoral evaluation) associated with photographs, cone-beam computed tomography, and plaster models of dental arches.

For planning, six facial features helped to plan for alteration of OP in orthognathic surgery: (I) facial type; (II) upper incisor display at rest regards to upper lip; (III) dental and gingival display on smile; (IV) fullness and soft tissue support in the lips and paranasal region; (V) chin projection regarding to lips; and (VI) lower lip projection.

**Figure 3:**
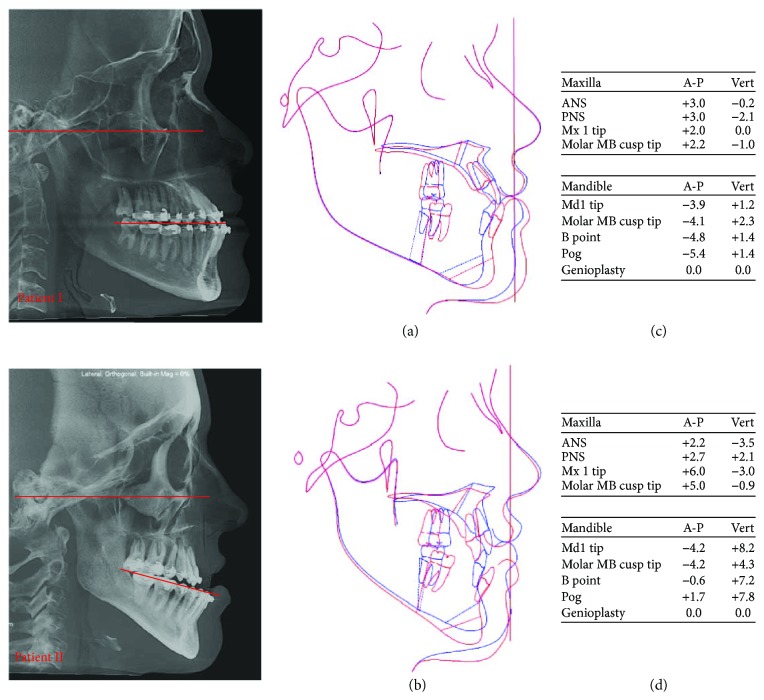
Illustrations of the lateral radiographs and red lines showed a qualitative comparison of OP inclination. (a-b) Superimpositions of original and predictive tracings. (c-d) Surgical movements.

The clinical facial characteristics of the patients were observed in a natural head position and properly registered (picture 1), correlating with the three-dimensional reconstructions of soft tissue and facial bone from the importation of DICOM (Digital Imaging and Communication in Medicine) from cone-beam computed tomography to Dolphin Imaging 11.7 Premium software (Dolphin Imaging and Management Solutions, Chatsworth, CA, USA). This software provided lateral radiographies and cephalometric measures that allowed bidimensional evaluation of the inclination of OP regards to Frankfort horizontal plane (Figure 3).

The plannings were based on the six clinical characteristics to obtain a harmony in facial appearance, considering patients' esthetic and functional complaints. Profile radiographs were performed for designing of original and predictive tracings (Figure 3) in a bidimensional evaluation. According to these factors, bimaxillary orthognathic surgeries were planned for both patients performed in an inverted sequence (mandible first). For patient I, it was planned alteration of OP in clockwise rotation of the MMC to decrease chin projection, to fill paranasal region, and to soften the mandibular contour. For patient II, it was planned alteration of OP of the MMC in counterclockwise rotation in order to improve chin posture in relation to the lower lip and to optimize the mandibular contour.

A conventional workflow was performed (wax bite registration under centric relation, facebow registration, and transfer of facebow registration to the semiadjustable articulator and model surgery). The surgical treatment plannings were simulated in model surgery, and the resulting postoperative model relationships were used to fabricate the intermediate and final splints. Theses splints were essential means to transfer the preoperative surgical treatment into accurate surgical procedure.

#### 2.2.2. Surgical Procedure

In both patients, the surgical procedures were performed under general anesthesia. Initially, buccal access to the mandible was achieved through soft tissue incision on the external oblique line to the mesial aspect of the second molar laterally (a minimum of 5 mm of nonkeratinized mucosa maintained in the buccal region). Subperiosteal dissection of the buccal mucosa was then performed towards the internal oblique line in the retromolar region, aiming at partially exposing the medial region and lingula of the mandible. Using reciprocating saws (Stryker-CORE System), sagittal osteotomy of the bilateral mandible was performed and finished using chisels. The intermediate splint was then fixed to the orthodontic appliance for maxillomandibular splinting with steel wire. The mandible and maxilla were stabilized in intermediate occlusion, and the mandible was repositioned via rigid internal fixation with straight miniplates and monocortical (System 2.0—Neoface—Neoortho Orthopedic Products).

Surgical access to the maxilla was performed buccally and subperiosteal nonkeratinized mucosa detachment, extending from the floor of the nasal fossa to the pterygomaxillary region. Le Fort I osteotomy was performed using a reciprocating saw (Stryker-CORE System) and finished with chisels. After osteotomy in the pterygomaxillary regions and mobilization of the maxilla, the walls of the maxilla were leveled following the planning, using rougher forceps and rotatory burs. The final splint was inserted along with the orthodontic appliances and steel wire to stabilize the maxilla and the mandible in final occlusion. Finally, the maxilla was repositioned with rigid internal fixation using L-shaped miniplates in the zigomaticomaxillary regions and around the pyriform aperture (System 2.0—Neoface—Neoortho Orthopedic Products). Mentoplasty was performed for chin advancement to improve the contour of the mentolabial groove. The surgical procedures were considered according to the planning and without intercurrences.

#### 2.2.3. Postoperative

The patient was evaluated weekly for the first 2 months and monthly thereafter until the sixth month. Postoperative orthodontic treatment was maintained through to completion.

**Figure 4:**
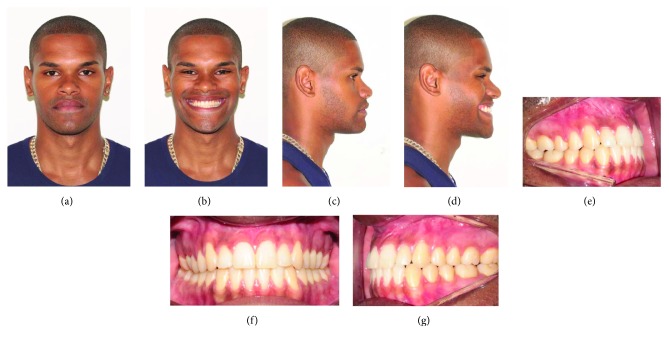
(a–d) Postoperative evaluation (2 years) at rest and smiling. (e–g) Intraoral images, postoperative occlusion after surgical and orthodontic treatment.

**Figure 5:**
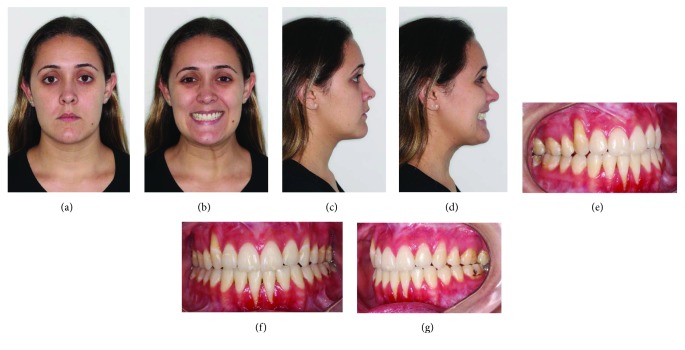
(a–d) Postoperative evaluation (2 years) at rest and smiling. (e–g) Intraoral images, postoperative occlusion after surgical and orthodontic treatment.

**Figure 6:**
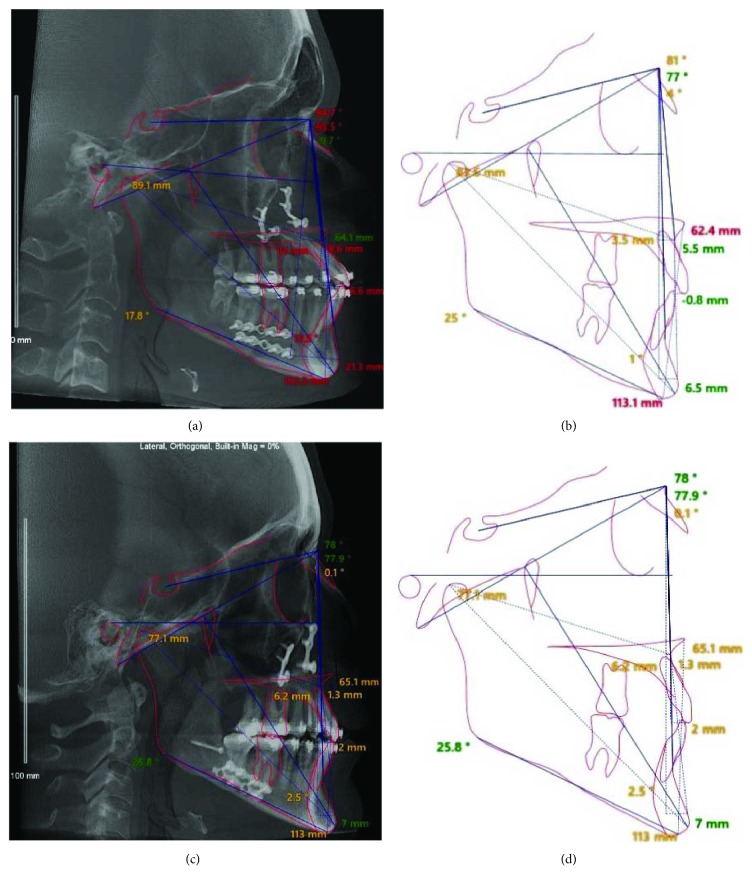
Illustrations of postoperative radiographs and lateral cephalometric. (a and b) Patient I. (c and d) Patient II.

Subjectively, the patients were satisfied with optimal esthetic and functional result. After 2 years of postoperative, the outcomes of the patients I and II showed, respectively, alterations of OP by clockwise (Figures 4(a)–4(g) and counterclockwise rotations of the MMC (Figures 5(a)–5(g)). The postoperative radiographies, lateral cephalometric tracings, and measures (McNamara Analysis) were presented at Figure 6 and Table 2, respectively.

## 3. Discussion

The bimaxillary orthognathic surgeries in both patients had been performed by alterations of OP, which proved to be a useful tool of planning to obtain favorable results. Thus, actual outcomes of Class III patients were based on planning and facial analysis of individual clinical features. The treatment planning of patients I and II was also based on the inclination of OP regarding to the Frankfort horizontal plane, but it was just qualitatively evaluated by comparing the posture of OP (Figure 3). Suchlike Parente et al. [14], similar planning to patients I and II were optimized by the clinical perception of the surgeons in detriment to the use of cephalometric tracings. Since 1993, Arnett and Bergman [15] had shown that performing orthognathic surgery planning only by cephalometric analysis could generate unfavorable esthetic results. Therefore, the planning cannot be based exclusively on cephalometric standards, as it could cause unfavorable esthetic results.

We also believe that lateral cephalometric analysis just quantifies dentoskeletal relationships in angular and linear measures (Tables 1 and 2). On the other hand, it cannot determine treatment planning in orthognathic surgery, because these measurements do not take into account the resting and dynamic relationships between hard and soft tissues, which are most critical aspects in treatment planning. Although the shortcoming of lateral cephalogram was determinant to not use as treatment goal, our case reports just were based on inclination of OP, there is matching with analysis of the facial morphologic form, soft tissue envelope, and the underlying facial bones integrated with dentition.

For Posnick et al. [16], facial esthetics can be achieved by changes in OP by counterclockwise or clockwise rotation of the MMC but emphasized that it is not a central point to quantify angular measurements of OP in the pre or post operatives, being more valid esthetic optimization by simply obtaining the most harmonic relations between skeletal structures and disposition of soft facial tissues. Marlière et al. [13] reinforced the idea that there was not an advantage to obtain the value of OP angulation, because OP angles may present wide variability in the population, there being more important to treatment planning based on surgeon's perceptions and clinical characteristics of each patient.

In this sense, six clinical characteristics observed in the patients were determinants for the planning of the surgical procedure. The facial type determined the way of OP alteration that was allowed by means of orthognathic surgery and counterclockwise rotation or clockwise rotation of the MMC. For patient I, clockwise rotation provided an increase in mandibular angle, and then, facial contour became more harmonic and soften (Figure 4). When the OP change was set in counterclockwise rotation, as performed in patient II, there was a decrease in the mandibular angle and the facial contour was improved (Figure 5). The upper incisor display regards to upper lip margins were evaluated at rest and on smile that had managed the anterior vertical repositioning of the maxilla. For it, 3 mm was acceptable to display upper incisors, and on smile, gingival display regarding to bordering the cervical gingival contour of the upper incisors, through to 2 mm were considered harmonious [17]. The soft tissue support was evaluated in lips and paranasal region, due to the maxillary advancement to improve the upper lip support and to provide paranasal fullness, so both treatment planning was adequately sufficient to achieve appropriate soft tissue support. The chin projection regarding lower incisor inclination helped to regulate the amount of OP alteration; because we believe higher discrepancy between these structures, greater alteration of OP would be necessary. Therefore, patient I benefited from clockwise rotation because the chin rotated posteriorly, and patient II was favored from counterclockwise rotation for the chin rotated anteriorly. Lower lip projection was properly achieved for both patients after OP alterations, which got a better chin position regards to lower lip, without needing for genioplasty and more natural outcomes. The clinical features of orthognathic surgery were also described by Marlière et al. [13] and Parente et al. [14], who were successful in planning and achieved satisfactory esthetic and functional results.

After diagnosis, facial analysis, and planning, bimaxillary orthognathic surgeries were performed based on mandible first sequence, starting from sagittal osteotomy of the mandible bilaterally. According to Borba et al. [18], orthognathic surgery in the inverted sequence approach was described in the 1970s. However, to date, the decision regarding such mandible first is based on the experience and preference of the surgeon. In addition, the inverted surgical sequence would be beneficial in situations such as clockwise rotation of the MMC to avoid an anterior open bite intraoperatively (when intermaxillary fixation is impaired by a thick intermediate guide), inaccuracy of intercondylar registration, and uncertainty regarding condylar positioning [17]. Borba et al. [18] also highlighted that the inverted sequence might not be preferred in surgical movements with clockwise rotation of the MMC, because rotations using posterior maxillary intrusion or anterior maxillary extrusion would require the mandible to be fixed in an “open bite” intermediate position with a thick intermediate splint in the incisor region, making the application of intermaxillary fixation difficult [19]. Another disadvantage, in cases undergoing the mandible first sequence in which an unfavorable split of the mandible occurs that is not correctable will have to postpone until a later date [20].

In terms of selection of surgical sequence, the inverted sequence of bimaxillary orthognathic surgery offered acceptable outcomes in patients I and II, regardless of whether clockwise rotation or counterclockwise rotation of the MMC. The traditional sequence of bimaxillary orthognathic surgery (maxilla first) was not preferred in surgical movements with clockwise rotation of the MMC, and we believe that the decision regarding which segment should be operated on first had relied on accurate preoperative planning based upon individual surgeon experience and preference.

A systematic review and meta-analysis published in 2016 compared postsurgical skeletal stability between counterclockwise and clockwise rotation of the MMC for correction of DFD. From screening and eligibility, three available studies were reviewed and showed that counterclockwise and clockwise rotations of the MMC are stable outcomes immediately after surgery and at longest follow-up, with no statistically significant difference between treatment planning, mainly, when there is no preexisting temporomandibular joint pathology [12]. Both Class III patients had similar skeletal stability because the postoperative outcomes have remained stable regarding facial esthetic and occlusal functionality in a follow-up over 2 years. Perhaps, they had treated by different planning based on alteration of OP from bimaxillary orthognathic surgery.

Finally, Class III patients had undergone same surgical treatment for correction of DFD, but different clinical features and inclination of OP helped to define treatment planning by clockwise rotation or counterclockwise rotation of the MMC. The clockwise and counterclockwise rotations of the MMC, also known as alteration of OP, should be considered to achieve soft tissue harmony among the subnasal, upper lip and lower lip support, and chin, because it influenced underlying facial skeleton integrated with the dental structures. These case reports showed that stable and harmonic outcomes between facial esthetics and occlusion are possible to achieve combining surgeon's clinical perception and qualitative evaluation of OP inclination, mainly, patients without facial asymmetry, because bidimensional images can represent the inclination bilaterally. Subjectively, outcomes after longest follow-up were associated with high patient satisfaction.

## Figures and Tables

**(a):** 

Quantitative and qualitative data from facial analysis		
Patient	Patient I Figures 1(a)–1(g)	Patient II Figures 2(a)–2(g)
Facial type (OP type)	Brachycephalic (low OP)	Dolichocephalic (high OP)
Upper incisor at rest	0 mm	7 mm
Dental and gingival display on the smile (mm^∗^)	Vertical maxillary deficiency 7 mm e 0 mm	Vertical maxillary excess 13 mm e 3 mm
Maxillary dental midline to the midsagittal plane	Dental midline shifted to the left	Dental midline to the right
Paranasal fullness	Paranasal fullness little decreased	Paranasal fullness decreased
Upper lip support (Nasolabial angle)	Good upper lip support (normal)	Lack of upper lip support (obtuse)
Display among soft tissue of lips and chin	Chin forward of upper and lower lips	Lower lip forward upper lip and chin
Malocclusion Reverse overjet/overbite (mm^∗^)	Class III −3 mm/0 mm	Class III −9 mm/0 mm

**(b):** 

Lateral cephalometric			
Skeletal sagittal relationship	Preoperative measurements		
	Patient I	Patient II	Range reference
SNA Angle	92.4°	75.5°	83.9° (±3.2°)
SNB Angle	94.3°	78.4°	81° (±3°)
ANB Angle	−2°	−2.9°	2° (±2°)
Point A to NPerp line	11.2 mm	−1.5 mm	1.1 mm (±2.7)
Pogonion to NPerp line	26.2 mm	4.2 mm	−0.3 mm (±3.8)
Mandible plane angle	18.4°	32.5°	21.3° (±3.9°)
Facial axis angle	10.8°	−1.9°	0.5 (±3.5°)
Maxilla incisor to point A	10.6 mm	3.4 mm	5.3 mm (±2)
Mandibular incisor to A-pogonion	8.7 mm	6.7 mm	2.3 mm (±2.1)

mm: millimeters; Nperp line: N perpendicular line.

**Table 2:** Comparison of postoperative cephalometric measures.

Lateral cephalometric			
Skeletal sagittal relationship	Postoperative measurements		
	Patient I	Patient II	Range reference
SNA angle	94.7°	81°	83.9° (±3.2°)
SNB angle	95.5°	77°	81° (±3°)
ANB angle	−0.7°	4°	2° (±2°)
Point A to NPerp line	9.6 mm	5.5 mm	1.1 mm (±2.7)
Pogonion to NPerp line	21.3 mm	6.5 mm	−0.3 mm (±3.8)
Mandible plane angle	17.8°	25°	21.3° (±3.9°)
Facial axis angle	13.5°	1°	0.5 (±3.5°)
Maxilla incisor to point A	13 mm	3.5 mm	5.3 mm (±2)
Mandibular incisor to A-pogonion	6.6 mm	−0.8 mm	2.3 mm (±2.1)

mm: millimeters; Nperp line: N perpendicular line.
